# Hematological Profiles and Serum Cytokine Levels in Patients With Breast Cancer in Low‐ and Middle‐Income Countries: A Systematic Review and Meta‐Analysis of Current Evidence

**DOI:** 10.1002/hsr2.73451

**Published:** 2026-10-27

**Authors:** Richard Vikpebah Duneeh, Otchere Addai‐Mensah, Benedict Sackey, Ernest Adankwah, Edward Yaw Afriyie, Veronica Agyemang, Precious Kwablah Kwadzokpui, Emmanuel Ativi, Mercy Adzo Klugah, Francis Agyei Amponsah, Kenneth Ablordey

**Affiliations:** ^1^ Department of Medical Laboratory Sciences, School of Allied Health Sciences University of Health and Allied Sciences Ho Ghana; ^2^ Department of Medical Diagnostics, Faculty of Allied Health Sciences, College of Health Sciences Kwame Nkrumah University of Science and Technology Kumasi Ghana; ^3^ Medical Laboratory Department Ho Teaching Hospital Ho Ghana; ^4^ Department of General and Liberal Studies, School of Basic and Biomedical Sciences University of Health and Allied Sciences Ho Ghana

**Keywords:** breast cancer, hematological parameters, LMICs, serum cytokines

## Abstract

**Background and Aim:**

Breast cancer is a leading cause of morbidity and mortality in Low‐ and Middle‐Income Countries (LMICs). Hematological and inflammatory markers offer accessible, low‐cost tools for patient assessment; hence, this study synthesized evidence on hematological parameters and serum cytokine levels in breast cancer patients from LMICs.

**Methods:**

Following PRISMA guidelines, we searched PubMed, Scopus, and Google Scholar for studies published between January 2013 and December 2025 comparing hematological parameters or serum cytokine levels between breast cancer patients and controls in LMICs, the protocol was registered on PROSPERO (CRD420261543115). Of 25 eligible studies identified, 22 were included in the meta‐analysis. Data on complete blood count indices and inflammatory cytokines were extracted and pooled using random‐effects models. Pooled effect sizes were calculated as standardized mean differences (SMD) with 95% confidence intervals. Subgroup analyses were performed by treatment status, and heterogeneity, sensitivity, and publication bias were assessed. All statistical analyses were performed using R software (version 4.5.0).

**Results:**

Erythrocyte parameters were consistently lower, including red blood cell count (SMD = −1.83; 95% CI: −3.32 to −0.33), packed cell volume (SMD = −4.04; 95% CI: −8.08 to −0.00), mean corpuscular volume (SMD = −1.15; 95% CI: −2.64 to −0.34), and mean corpuscular hemoglobin (SMD = −0.99; 95% CI: −2.19 to −0.20). Platelet‐related parameters were elevated, including pretreatment platelet count (SMD = 0.44; 95% CI: 0.17–0.70) and mean platelet volume (SMD = 0.44; 95% CI: 0.07–0.81). Leukocyte profiles showed decreased lymphocytes (SMD = −1.07; 95% CI: −1.82 to −0.83) and eosinophils (SMD = −2.33; 95% CI: −5.90 to −1.25), with increased monocytes (SMD = 0.79; 95% CI: 0.29– 1.29). One study reported markedly elevated inflammatory cytokines in patients versus controls: IL‐6 (20.6 vs. 4.4 pg/mL), IL‐10 (8.8 vs. 2.8 pg/mL), and TNF‐α (26.0 vs. 4.7 pg/mL); all *p* = 0.001.

**Conclusion:**

These findings support the integration of routine complete blood count parameters as accessible, low‐cost tools for monitoring disease burden and systemic inflammation in resource‐limited settings. Further research is needed to characterize cytokine profiles and establish normative reference data for LMIC populations.

## 1 Introduction

Breast cancer remains a leading cause of cancer‐related morbidity and mortality worldwide and poses a substantial and growing public health challenge in low‐ and middle‐income countries (LMICs), particularly in Africa [1]. Increasing incidence rates, late‐stage presentation, and limited access to advanced diagnostic and monitoring technologies contribute to disproportionately high mortality in these settings [2]. In resource‐constrained environments, reliance on affordable, readily available laboratory investigations is often necessary, underscoring the importance of identifying accessible biomarkers that may assist in disease assessment and monitoring.

Complete blood count (CBC) is one of the most common blood tests requested by clinicians and evaluates the total numbers and characteristics of cell components in the blood [3, 4]. Alterations in hemoglobin concentration, red blood cell (RBC) indices, platelet parameters, and leukocyte subsets have been reported in breast cancer patients and are thought to reflect cancer‐associated anemia, systemic inflammation, immune dysregulation, and treatment‐related effects [5, 6, 7]. Anemia may result from chronic inflammation, nutritional deficiencies, bone marrow suppression, or chemotherapy, while changes in platelet and leukocyte profiles may mirror tumor‐driven inflammatory and immune responses [8, 9]. These hematological alterations, therefore, may provide indirect insight into tumor biology and host response.

Inflammatory cytokines further play a critical role in breast cancer pathophysiology. Within the tumor microenvironment, cytokines regulate angiogenesis, cellular proliferation, invasion, and immune evasion [10]. Elevated circulating cytokine levels have been associated with persistent inflammation and tumor progression, suggesting their potential value as indicators of disease activity and immune imbalance. Importantly, systemic inflammatory responses mediated by cytokines may also contribute to observable changes in peripheral blood indices, linking immune signaling pathways with hematological alterations [11, 12].

Despite growing evidence, studies from LMICs examining hematological parameters and cytokine profiles in breast cancer remain limited and heterogeneous, with inconsistent findings across populations. A comprehensive synthesis of these biomarkers in LMIC settings is lacking. Therefore, this systematic review and meta‐analysis aimed to evaluate and integrate available evidence on hematological indices and serum cytokine levels in breast cancer patients from LMICs, with the objective of confirming their potential utility as accessible adjunctive tools for disease monitoring and clinical management in resource‐limited settings, while identifying gaps to guide future research.

## 2 Methods and Materials

### 2.1 Study Area

The research focused exclusively on the LMICs from January 2013 to December 2025.

### 2.2 Study Design/Protocol

This research employed a systematic literature review to meticulously gather and analyze existing data, with the primary aim of establishing the mean difference of hematological and serum cytokine levels in breast cancer patients and controls. To ensure methodological transparency and adherence to best practices, the study adhered rigorously to the guidelines set forth by the Preferred Reporting Items for Systematic Reviews and Meta‐Analyses (PRISMA) framework. The protocol for the development of this systematic review and meta‐analysis was registered on the International Prospective Register of Systematic Reviews (PROSPERO) under reference number (CRD420261543115).

### 2.3 Criteria for Considering Studies for This Review

#### 2.3.1 Types of Studies

This systematic review incorporated case‐control studies, as well as prospective or retrospective cohort studies, exclusively centered on reporting the hematological profile and serum cytokines in breast cancer patients. The selection encompassed scholarly journal articles published as primary research studies. The timeframe for inclusion ranged from January 2013, to December, 2025, exclusively within any country classified as LMIC by the World Bank Group in 2020 (i.e., LMICs are categorized into low‐income countries [$1045 or less], low‐middle‐income [$1046–$4095], and upper‐middle‐income [$4096–$12,695] [13], studies that focused on breast cancer, studies published in peer‐reviewed journals and studies published in the English language to capture cytokines and hematological parameters in breast cancer. Editorials, opinion pieces, comments, letters, reviews, and studies focused on high‐income settings were excluded. See Supporting Information S1 for details on classification of LMIC.

#### 2.3.2 Participant Profile

This study included participants diagnosed with breast cancer in LMICs, as reported in eligible studies. The selected studies comprised patients across different stages of breast cancer, with varying demographic, clinical, and pathological characteristics. Key variables assessed included age, gender, cancer stage, treatment status, and hematological parameters.

### 2.4 Search Methods for Identification of Studies

#### 2.4.1 Information Sources

Three electronic databases were searched, namely, Scopus, PubMed, and Google Scholar. A hand search of the reference lists of included studies was performed to supplement the database search.

#### 2.4.2 Search Strategy

Databases were searched from January 2013 to December 2025. The search strategy included terms relating to the following concepts: breast cancer, breast cancer patients, cytokines, serum cytokines, interleukin‐1 beta (IL‐1β), interleukin‐2 (IL‐2), interleukin‐6 (IL‐6), interleukin‐8 (IL‐8 or CXCL8), tumor necrosis factor‐alpha (TNF‐α), interferon‐gamma (IFN‐γ), interleukin‐4 (IL‐4), interleukin‐10 (IL‐10), interleukin‐13 (IL‐13), transforming growth factor‐beta (TGF‐β), interleukin‐17 (IL‐17), interleukin‐18 (IL‐18), interleukin‐12 (IL‐12), interleukin‐23 (IL‐23), interleukin‐22 (IL‐22), granulocyte‐macrophage colony‐stimulating factor (GM‐CSF), and vascular endothelial growth factor (VEGF), hematological profile/parameters, haemogram, full blood count (FBC), complete blood count (CBC), red blood cell (RBC), white blood cell (WBC), hemoglobin (HB), platelets (PLT), hematocrit (HCT), monocytes, lymphocytes, eosinophils, basophils, mean platelet value (MPV), mean cell volume (MCV), mean corpuscular hemoglobin (MCH), mean corpuscular hemoglobin concentration (MCHC) and LMICs. Medical subject headings, keywords, and free text terms were combined using “AND” or “OR” Boolean operators. The initial search strategy was developed in PubMed.

### 2.5 Data Collection and Analysis

#### 2.5.1 Study Selection

A total of 4521 records were identified through database searching, of which 1004 duplicates were removed before screening. The remaining 3517 records were screened based on titles and abstracts, leading to the exclusion of 1907 records. Full‐text assessment was conducted for 1610 articles, after which 1585 were excluded due to being editorials or reviews (*n *= 1011), non‐breast cancer studies (*n *= 521), or wrong study settings (*n *= 53). Ultimately, 25 studies met the inclusion criteria, of which 22 were included in the meta‐analysis (Figure 1). Two authors independently screened titles and abstracts of citations retrieved by the search for relevance against the inclusion criteria, and full texts of articles were obtained. A third author independently screened 10% of the articles. Microsoft Excel 2021 and Zotero referencing manager version 7 were used to manage citations and the screening process, including duplicate identification. Disagreements were resolved through discussion.

**Figure 1:**
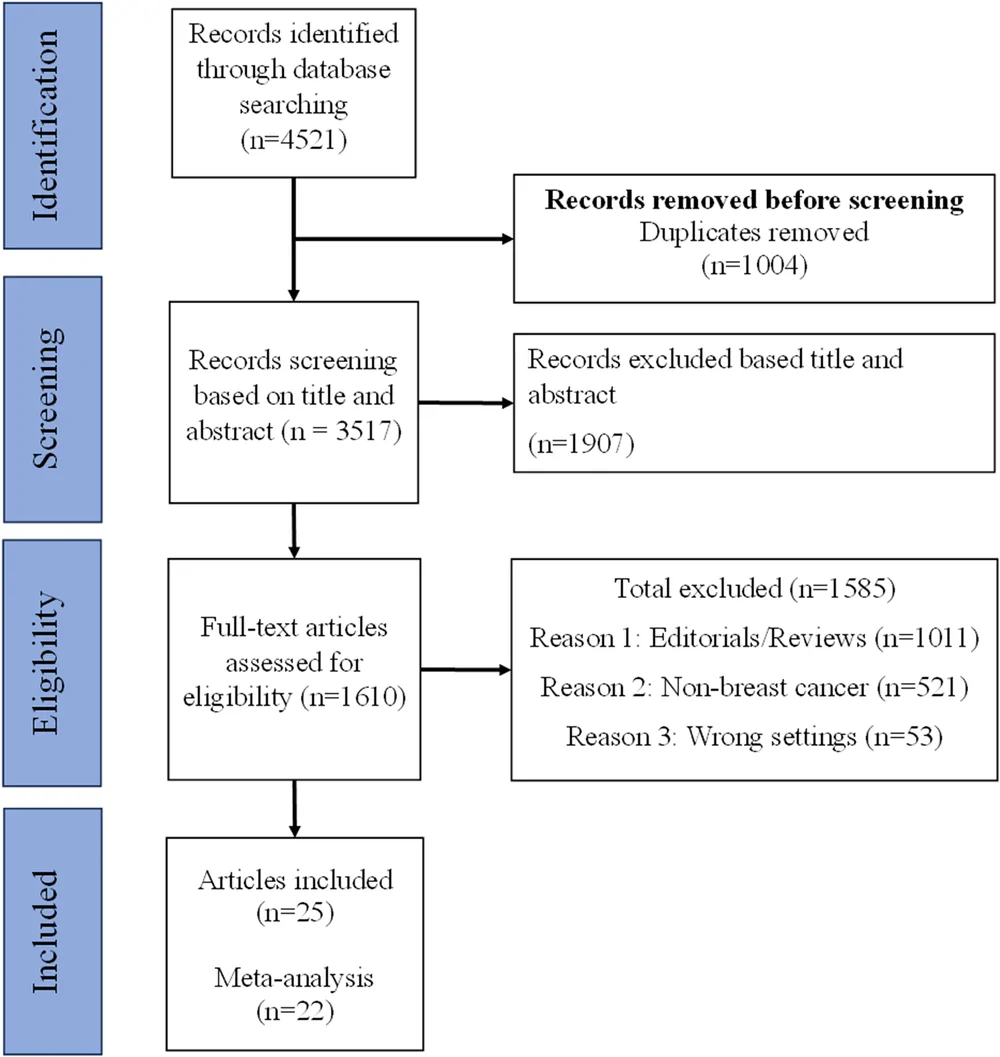
PRISMA flow diagram summarizing the identification, screening, eligibility assessment, and inclusion of studies in the systematic review and meta‐analysis.

#### 2.5.2 Data Extraction and Management

An electronic data extraction form was developed, and full‐text data extraction was performed by three authors. The extracted data was reviewed and discussed in a team meeting, and disagreements were resolved through consensus. Data relating to the characteristics of each study, such as the author and year of publication, study design, sample size, country of study, method of diagnosis, study period, and age range of participants, were extracted from each study. All data retrieved from the studies were entered into Microsoft Excel 2021. The first author's surname and the year of publication of the study were used as identities for each study. The sample size for both control and cases (breast cancer patients), mean, and standard deviation for both cases and controls were used for the meta‐analysis. For a parameter where only one study was obtained, the results of the study were tabulated for the purposes of discussion without meta‐analysis.

### 2.6 Statistical Analysis and Data Synthesis

Data were extracted from included studies and entered into a Microsoft Excel spreadsheet 2021, capturing author names, year of publication, country, study design, sample sizes, demographic characteristics, analyzer type, breast cancer confirmation method, cancer stage, treatment status, control type, and complete blood count with corresponding means and standard deviations for both breast cancer cases and control groups. All statistical analyses were performed using R software (version 4.5.0) with the meta and metafor packages. For each hematological parameter, the standardized mean difference with 95% confidence intervals was calculated as the effect size measure using Hedges' *g* correction for small sample bias to accommodate different measurement units and scales across studies. Random‐effects models were applied for all meta‐analyses using the restricted maximum likelihood method with Hartung–Knapp adjustment, accounting for both within‐study sampling error and between‐study heterogeneity while providing more adequate error rates and wider confidence intervals when the number of studies is small. For each meta‐analysis, we computed the pooled standardized mean difference, its 95% confidence interval, and the corresponding *p* value for the overall effect. Between‐study heterogeneity was assessed using Cochran's Q test with a *p* value less than 0.10 considered statistically significant, the *I* ^2^ statistic to quantify the proportion of total variation attributable to heterogeneity with values interpreted as 0%–40%; low, 30%–60%; moderate, 50%–90%; substantial, and 75%–100%; considerable heterogeneity, and the between‐study variance estimated using the restricted maximum likelihood method. Subgroup analyses were performed stratifying studies into before treatment and chemotherapy groups, with separate random‐effects meta‐analyses conducted for each subgroup and pooled standardized mean differences with 95% confidence intervals calculated only for subgroups with at least two studies. Sensitivity analyses were conducted using leave‐one‐out methods to evaluate the robustness of pooled effect sizes, with the recalculated pooled standardized mean difference and *I* ^2^ values recorded as ranges and robustness classified as robust if statistical significance remained unchanged after removal of any single study and unstable if exclusion altered significance; parameters with fewer than three studies were excluded from leave‐one‐out sensitivity analysis. Publication bias was assessed for parameters with at least three studies using visual inspection of contour‐enhanced funnel plots, Egger's linear regression test and Begg's rank correlation test with a *p* value less than 0.10 considered indicative of potential publication bias, and trim‐and‐fill analysis to estimate the impact of potential publication bias by imputing hypothetical missing studies and reporting the number of missing studies, adjusted standardized mean difference, and adjusted 95% confidence interval for each parameter. Forest plots were generated for all primary meta‐analyses to display individual study effect sizes, 95% confidence intervals, study weights, and the pooled random‐effects estimate with confidence and prediction intervals. A *p* value less than 0.05 was considered statistically significant for pooled effect estimates, while for heterogeneity and publication bias tests a threshold of *p* less than 0.10 was applied due to the conservative nature of these tests with small numbers of studies.

### 2.7 Methodological Quality Assessment of Included Studies Using the Newcastle–Ottawa Scale

The methodological quality of the included studies was independently assessed by two reviewers (K.A. and E.A.) using the Newcastle–Ottawa Scale (NOS) for case‐control and cohort studies (Table 1) [14] while the study selection process was done in accordance with PRISMA guidelines (Figure 1). Overall, the methodological quality of the included studies was moderate. Total NOS scores ranged from 3 to 6 out of a possible 9 points. Most studies achieved satisfactory ratings in the selection domain, reflecting appropriate case definitions and control selection in several reports. However, comparability was variably addressed, with fewer studies clearly demonstrating adjustment for important confounders. Similarly, while many studies adequately described exposure or outcome ascertainment, incomplete reporting in some articles limited higher scoring in this domain. Collectively, although the majority of studies were of acceptable quality, methodological limitations, particularly regarding control of confounding and reporting transparency, were present.

**Table 1:** Methodological quality assessment of included studies using the Newcastle–Ottawa scale.

Authors	Selection					Outcome/exposure			Total score
	Item 1	Item 2	Item 3	Item 4	Comparability	Item 1	Item 2	Item 3	
Aynalem et al. [6]			*			*	*		3
Divsalar et al. [15]	*			*	*	*	*		5
Manigatta et al. [16]	*			*		*	*		4
Ali [17]	*		*	*		*	*		5
Danish et al. [18]	*		*	*	*	*	*		6
Khan et al. [19]	*			*	*	*	*		5
Udosen et al. [20]	*			*		*	*		4
Ogochukwu et al. [21]	*			*	*	*	*		5
Rana et al. [22]	*			*		*	*		4
Akanni [5]	*								
Masamatti [23]		*	*		*		*		4
Kalila [24]	*				*		*		3
Abbas et al. [25]		*		*	*				3
Osaro et al. [26]		*		*	*		*		4
Ahmed et al. [27]	*		*		*	*			4
Echonwere‐uwikor and Uwikor [28]			*		*		*		3
Ngozi et al. [29]	*		*		*		*		4
Abedalrahman et al. [30]	*			*	*				3
Chinedu‐madu et al. [7]		*		*	*		*		5
Storph et al. [31]	*		*	*			*		4
Shrivastava et al. [32]		*		*	*		*		4
Nnenna et al. [33]	*	*		*	*		*		5
Clara et al. [34]	*		*	*	*	*			4
Akinbami et al. [35]				*	*	*			3
Hussain et al. [36]	*			*		*	*		4

*Note:* Quality assessment of included studies according to the Newcastle–Ottawa Scale (NOS). The NOS evaluates methodological quality across three domains: selection (four items), comparability (two items), and exposure/outcome (three items). Asterisks (*) indicate fulfillment of individual checklist criteria. Higher total scores reflect better methodological quality.

## 3 Results

### 3.1 Characteristics of Included Studies

A total of 25 studies published between 2013 and 2025 were included in this review, the majority of which employed a case‐control design. The studies were conducted predominantly in low‐ and lower‐middle‐income countries, with Nigeria, India, Iraq, and Iran contributing the largest number of reports. Only one retrospective cohort study [6] and one cross‐sectional study [31] were identified, while all remaining studies were case‐control in design. Breast cancer diagnosis was confirmed using histological methods in four studies [29, 30, 37, 38], cytological examination in one study [23], and mammography in one study [36], whereas several studies did not explicitly state the method of confirmation. Most studies evaluated patients either before treatment initiation or during chemotherapy, with stage I–IV disease commonly represented when staging was reported. Automated hematology analyzers, particularly Sysmex platforms (e.g., KX‐21N, XP‐300, XN series), were predominantly used for laboratory measurements, although a few studies employed other automated systems or manual methods. Controls were generally healthy individuals, although some studies included benign breast disease patients as comparators (Table 2).

**Table 2:** Characteristics of included studies.

Authors	Year of publication	Country	Type of income country	Study design	Total sample size	Sample size (case)	Sample size (control)	Type of analyzer	Breast cancer confirmation	Stage of breast cancer	Treatment	Type of control
Aynalem et al. [6]	2022	Ethiopia	Low‐income	Retrospective cohort	267	267	—	—	—	I‐IV	Before treatment	—
Divsalar et al. [15]	2021	Iran	Upper‐middle‐income	Case control	320	160	160	Sysmex Kx‐21n	—	—	—	Healthy
Shilpa et al. [16]	2020	India	Lower‐middle‐income	Case control	90	70	30	Sysmex	—	I‐IV	Before treatment	Benign
Ali [17]	2014	Iraq	Upper‐middle‐income	Case control	50	30	20	Auto haematology analyzer, hungry 2011	—	—	—	Healthy
Danesh et al. [39]	2022	Iran	Upper‐middle‐income	Case control	80	43	37	Sysmex KX‐21N	—	—	Before treatment	Healthy
Khan et al. [19]	2017	Pakistan	Lower‐middle‐income	Case control	120	80	40	—	—	—	—	Healthy
Udosen et al. [20]	2022	Nigeria	Lower‐middle‐income	Case control	66	36	30	Sysmex Kx 21n	—	—	Chemotherapy	Healthy
Izuegbuna et al. [37]	2020	Nigeria	Lower‐middle‐income	Case control	65	15	50	Sysmex KN‐ 21N haemato‐ analyzer	Histological	I‐IV	Before treatment	Healthy
Rana et al. [22]	2015	India	Lower‐middle‐income	Case control	50	35	15	—	Histological	I‐IV	Chemotherapy	Benign
Akanni [5]	2014	Iraq	Upper‐middle‐income	Case control	88	66	22	—	—	I‐III	Before treatment	Healthy
Masamatti [23]	2018	India	Lower‐middle‐income	Case control	102.00	51	51	Sysmex XN 550	Cytological	—	Chemotherapy	Benign
Kalila [24]	2024	Libya	Upper‐middle‐income	Case control	200	100	100	Sysmex (KX 21)	NS	—	—	Healthy
Abbas et al. [25]	2024	Yemen	Low‐income	Case control	200	100	100	—	—	—	—	Healthy
Osaro et al. [26]	2018	Nigeria	Lower‐middle‐income	Case control	70	24	46	Auto haematology analyzer (genesis HA6000, perlong, China)	—	I‐IV	Chemotherapy	Healthy
Ahmed et al. [27]	2025	Sudan	Low‐income	Case control	100	50	50	Sysmex XP‐300	—	NS	Chemotherapy	Healthy
Echonwere‐Uwikor and Uwikor [28]	2024	Nigeria	Lower‐middle‐income	Case control	410	310	100	Sysmex XN‐330	—	I‐IV	Chemotherapy	Healthy
Ngozi et al. [29]	2025	Nigeria	Lower‐middle‐income	Case control	125	50	75	Sysmex XP‐300	Histological	I‐IV	—	Healthy
Abedalrahman et al. [30]	2022	Iraq	Upper‐middle‐income	Case control	100	50	50	—	Histological	I‐IV	—	Benign
Chinedu‐Madu et al. [7]	2025	Nigeria	Lower‐middle‐income	Case control	40	20	20	Hemocytometer	—	—	—	Healthy
Storph et al. [31]	2019	Ghana	Lower‐middle‐income	Cross‐sectional	6	6	—	—	—		Before treatment	
Shrivastava et al. [32]	2017	India	Lower‐middle‐income	Case control	151	51	100	Sysmex (KX‐21)	—	I‐IV	Chemotherapy	Healthy
Nnenna et al. [33]	2024	Nigeria	Lower‐middle‐income	Case control	120	60	60	Mindray BC‐5180	—	I‐IV	—	Healthy
Clara et al. [34]	2022	Nigeria	Lower‐middle‐income	Case control	220	110	110	Mindray Be‐5300	—	—	Before treatment	Healthy
Akinbami et al. [35]	2013	Nigeria	Lower‐middle‐income	Case control	150	100	50	Sysmex KN‐21N	—	—	Before treatment	Healthy
Hussain et al. [36]	2020	Iraq	Upper‐middle‐income	Case control	80	20	60	ELISA	Mammography		Before treatment	Healthy

*Note:* Summary characteristics of the included studies evaluating hematological parameters in breast cancer patients. Information includes country and income classification, study design, sample size, laboratory analyzer used, method of breast cancer confirmation, disease stage, treatment status, and type of control group.

### 3.2 Hematological and Inflammatory Parameters Assessed in the Included Studies

The majority of studies assessed core red cell indices, including hemoglobin (Hb), red blood cell count (RBC), hematocrit/packed cell volume (HCT/PCV), mean corpuscular volume (MCV), mean corpuscular hemoglobin (MCH), and mean corpuscular hemoglobin concentration (MCHC). Total white blood cell (WBC) count and differential leukocyte parameters, particularly neutrophil and lymphocyte counts, were also frequently reported, whereas monocyte and eosinophil counts were evaluated in a smaller number of studies [19, 20, 25, 32, 33]. Platelet count was examined in most studies, while red cell distribution width (RDW) and mean platelet volume (MPV) were assessed less consistently. Notably, one study focused exclusively on inflammatory biomarkers, specifically interleukin‐6 (IL‐6), interleukin‐10 (IL‐10), and tumor necrosis factor‐alpha (TNF‐α) [36] (Table 3).

**Table 3:** Hematological and inflammatory parameters assessed in the included studies.

Authors (references)	Parameters assessed
Aynalem et al. [6]	RBC, HB, HCT, MCV, MCH, MCHC, WBC, neutrophil, lymphocyte, platelets, RDW, MPV.
Divsalar et al. [15]	HB, HCT, MCV, MCH, MCHC, WBC, platelets, RDW, MPV
Shilpa et al. [16]	RBC, HB, HCT, MCV, MCH, MCHC, WBC, neutrophil, lymphocyte, platelets.
Ali [17]	WBC, RBC, PCV, platelets, lymphocyte
Danesh et al. [18]	HB, RBC, HCT, MCV, MCH, MCHC, WBC, platelets.
Khan et al. [19]	RBC, WBC, monocytes, neutrophils, lymphocytes.
Udosen et al. [20]	HB, PCV, WBC, platelets, neutrophil, lymphocyte, monocyte, eosinophil.
Izuegbna et al. [37]	HB, HCT, MCV, MCH, MCHC, WBC, platelets, RDW, MPV, PCV, lymphocyte, neutrophil.
Rana et al. [22]	RBC, HB, PCV, WBC, MCV, MCH, MCHC, platelets, neutrophil, lymphocyte, monocyte, eosinophil.
Akanni et al. [5]	RBC, HB, PCV, MCV, MCH, MCHC, WBC, platelets, neutrophil, lymphocyte,
Masamatti [23]	HB, PCV, MCV, MCH, MCHC, WBC, neutrophils, lymphocytes, platelets, RDW, MPV
Kalila [24]	RBC, HB, HCT, MCV, MCH, MCHC, WBC, neutrophils, lymphocytes, platelets.
Abbas [25]	RBC, HB, PCV, MCV, MCH, MCHC, WBC, eosinophils, monocytes, neutrophils, lymphocytes, platelets.
Osaro et al. [26]	HB, WBC, platelets.
Ahmed et al. [27]	RBC, WBC, platelets.
Echonwere‐Uwikor and Uwikor [28]	RBC, Hb, PCV, MCV, MCH, platelets.
Ngozi et al. [29]	HB, WBC, neutrophils, lymphocytes.
Abedalrahman et al. [30]	HB, PCV, WBC, neutrophils, lymphocytes, platelets, RDW, MPV.
Chinedu‐madu et al. [7]	RBC, MCV, MCH.
Storph et al. [31]	HB, platelets.
Shrivastava et al. [32]	RBC, HB, WBC, eosinophils, monocytes, neutrophils, lymphocytes
Nnenna et al. [33]	BC, HB, PCV, MCV, MCH, MCHC, WBC, eosinophils, monocytes, neutrophils, lymphocytes, platelets, RDW.
Clara et al. [34]	RBC, HB, PCV, MCV, MCH, MCHC, platelets, RDW, MPV.
Akinbami et al. [35]	MCV, MCH, MCHC, WBC, neutrophils, lymphocytes, platelets, RDW.
Hussain et al. [36]	IL‐6, IL‐10, TNF Alpha

*Note:* Summary of hematological and inflammatory parameters assessed by each included study.

Abbreviations: HB, hemoglobin; HCT, hematocrit; IL‐6, interleukin‐6; IL‐10, interleukin‐10; MCH, mean corpuscular hemoglobin; MCHC, mean corpuscular hemoglobin concentration; MCV, mean corpuscular volume; MPV, mean platelet volume; PCV, packed cell volume; RBC, red blood cell count; RDW, red cell distribution width; TNF‐α, tumor necrosis factor‐alpha; WBC, white blood cell count.

### 3.3 Publication Bias Assessment of Hematological Parameters

Evidence of small‐study effects was observed for RBC count, lymphocyte count, neutrophil count, MCHC, and platelet count based on Egger's and/or Begg's tests (*p *< 0.05). After trim‐and‐fill adjustment, RBC count (adjusted SMD = −1.81; 95% CI: −3.32 to −0.33) and lymphocyte count (adjusted SMD = −1.59; 95% CI: −3.19 to −0.02) remained significantly reduced in breast cancer patients, while neutrophil count remained significantly elevated (adjusted SMD = 1.11; 95% CI: 0.29–1.93), indicating that the direction and statistical significance of these associations were robust to potential publication bias. For hemoglobin, hematocrit/packed cell volume, MCV, MCH, WBC, eosinophils, monocytes, and MPV, neither statistical tests nor trim‐and‐fill adjustments materially altered the pooled effect estimates, suggesting no strong evidence of publication bias (Table 4).

**Table 4:** Publication bias assessment of hematological parameters.

Parameter	Studies	Egger's test		Begg's test		Trim‐and‐fill analysis		
		*t*	*p* value	*τ*	*p* value	Missing	Adj. SMD	Adj. 95% CI
Hb	15	−1.975	0.070	0.048	0.846	0	−2.31	[−5.46, 0.80]
RBC	15	−3.451	0.004	−0.448	0.021	6	−1.81	[−3.32, −0.33]
HCT/PCV	6	−1.287	0.267	0.067	1.000	1	−2.59	[−7.01, 1.78]
MCV	13	−1.302	0.220	−0.154	0.510	1	−1.15	[−2.64, 0.34]
MCH	13	−1.592	0.140	−0.154	0.510	1	−0.99	[−2.19, 0.20]
MCHC	11	−2.594	0.029	−0.127	0.648	3	−1.09	[−2.73, 0.54]
WBC	15	−0.201	0.844	−0.219	0.282	5	0.27	[−1.77, 2.28]
Lymphocyte	14	−2.836	0.015	−0.560	0.005	6	−1.59	[−3.19, −0.02]
Neutrophil	13	2.292	0.043	0.462	0.030	4	1.11	[0.29, 1.93]
Eosinophil	5	−2.47	0.090	−0.400	0.483	1	−2.3	[−5.90, 1.25]
Monocyte	6	0.924	0.408	0.200	0.719	1	0.79	[−0.28, 1.86]
PLT	16	−2.23	0.043	−0.367	0.052	5	−0.38	[−1.10, 0.33]
MPV	6	−0.765	0.487	−0.333	0.469	1	0.44	[0.07, 0.81]

*Note:* Assessment of publication bias for pooled hematological parameters using Egger's regression test, Begg's rank correlation test, and trim‐and‐fill analysis. “Missing” indicates the number of imputed studies under the trim‐and‐fill method, and “Adj. SMD” represents the adjusted standardized mean difference with corresponding 95% confidence interval (CI).

Abbreviations: CI, confidence interval; SMD, standardized mean difference.

### 3.4 Sensitivity Analysis of Hematological Parameters

Across outcomes, sequential exclusion of individual studies resulted in only modest variations in effect sizes, with pooled SMDs remaining within relatively narrow ranges and largely preserving their direction of effect. In particular, RBC count, lymphocyte count, neutrophil count, and mean platelet volume (MPV) consistently retained both the magnitude and direction of association observed in the primary analysis. Although heterogeneity remained high for most parameters (*I* ^2^ approximately 90%–100%), the *I* ^2^ values demonstrated minimal fluctuation during LOO analysis, indicating that no single study disproportionately influenced between‐study variability (Table 5).

**Table 5:** Sensitivity analysis of hematological parameters.

Parameter	Studies (*n*)	Cases (*n*)	Controls (*n*)	Original SMD [95% CI]	Original *I* ^2^ (%)	SMD range (LOO)	*I* ^2^ range (LOO)
Hb	15	1011	1048	−2.33 [−5.46, 0.80]	1.0	[−2.53, −0.76]	0.9–1.0
RBC	15	1004	972	−1.83 [−3.32, −0.33]	1.0	[−1.98, −1.12]	0.9–1.0
HCT/PCV	6	533	487	−2.62 [−7.01, 1.78]	1.0	[−3.27, −0.41]	0.9–1.0
MCV	13	1219	948	−1.15 [−2.64, 0.34]	1.0	[−1.29, −0.40]	0.9–1.0
MCH	13	1219	948	−0.99 [−2.19, 0.20]	1.0	[−1.12, −0.41]	0.9–1.0
MCHC	11	889	828	−1.09 [−2.73, 0.54]	1.0	[−1.25, −0.28]	0.9–1.0
WBC	15	998	928	0.25 [−1.77, 2.28]	1.0	[−0.62, 0.75]	1.0–1.0
Lymphocyte	14	953	956	−1.61 [−3.19, −0.02]	1.0	[−1.89, −0.87]	1.0–1.0
Neutrophil	13	952	876	1.11 [0.29, 1.93]	1.0	[0.76, 1.27]	0.9–1.0
Eosinophil	5	407	450	−2.33 [−5.90, 1.25]	1.0	[−2.91, −0.52]	0.9–1.0
Monocyte	6	487	490	0.79 [−0.28, 1.86]	1.0	[0.41, 1.13]	1.0–1.0
PLT	16	1338	1073	−0.39 [−1.10, 0.33]	1.0	[−0.48, −0.13]	0.9–1.0
MPV	6	446	481	0.44 [0.07, 0.81]	0.8	[0.35, 0.58]	0.7–0.8

*Note:* Leave‐one‐out (LOO) sensitivity analysis of pooled standardized mean differences (SMDs) for hematological parameters comparing breast cancer patients with controls. The SMD range and *I* ^2^ range reflect the variation in pooled estimates and heterogeneity after sequential exclusion of each individual study.

Abbreviations: Hb, hemoglobin; HCT, hematocrit; MCH, mean corpuscular hemoglobin; MCHC, mean corpuscular hemoglobin concentration; MCV, mean corpuscular volume; MPV, mean platelet volume; PCV, packed cell volume; PLT, platelet count; RBC, red blood cell count; WBC, white blood cell count.

### 3.5 Random‐Effects Meta‐Analysis of Hemoglobin, Red Blood Cell Count, Hematocrit, and Packed Cell Volume Among Breast Cancer Patients vs. Controls

Hemoglobin levels were lower among breast cancer patients, although the pooled effect did not reach statistical significance (SMD = −2.33; 95% CI: −5.46 to 0.80; *I* ^2 ^= 97.3%). In contrast, RBC count was significantly reduced in breast cancer patients (SMD = −1.83; 95% CI: −3.32 to −0.33; *I* ^2 ^= 97.6%). Hematocrit also showed a non‐significant reduction (SMD = −2.62; 95% CI: −7.01 to 1.78; *I* ^2 ^= 98.8%). Packed cell volume demonstrated a statistically significant decrease (SMD = −4.04; 95% CI: −8.08 to −0.004; *I* ^2 ^= 97.4%). Heterogeneity was consistently high across all outcomes (Figure 2).

**Figure 2:**
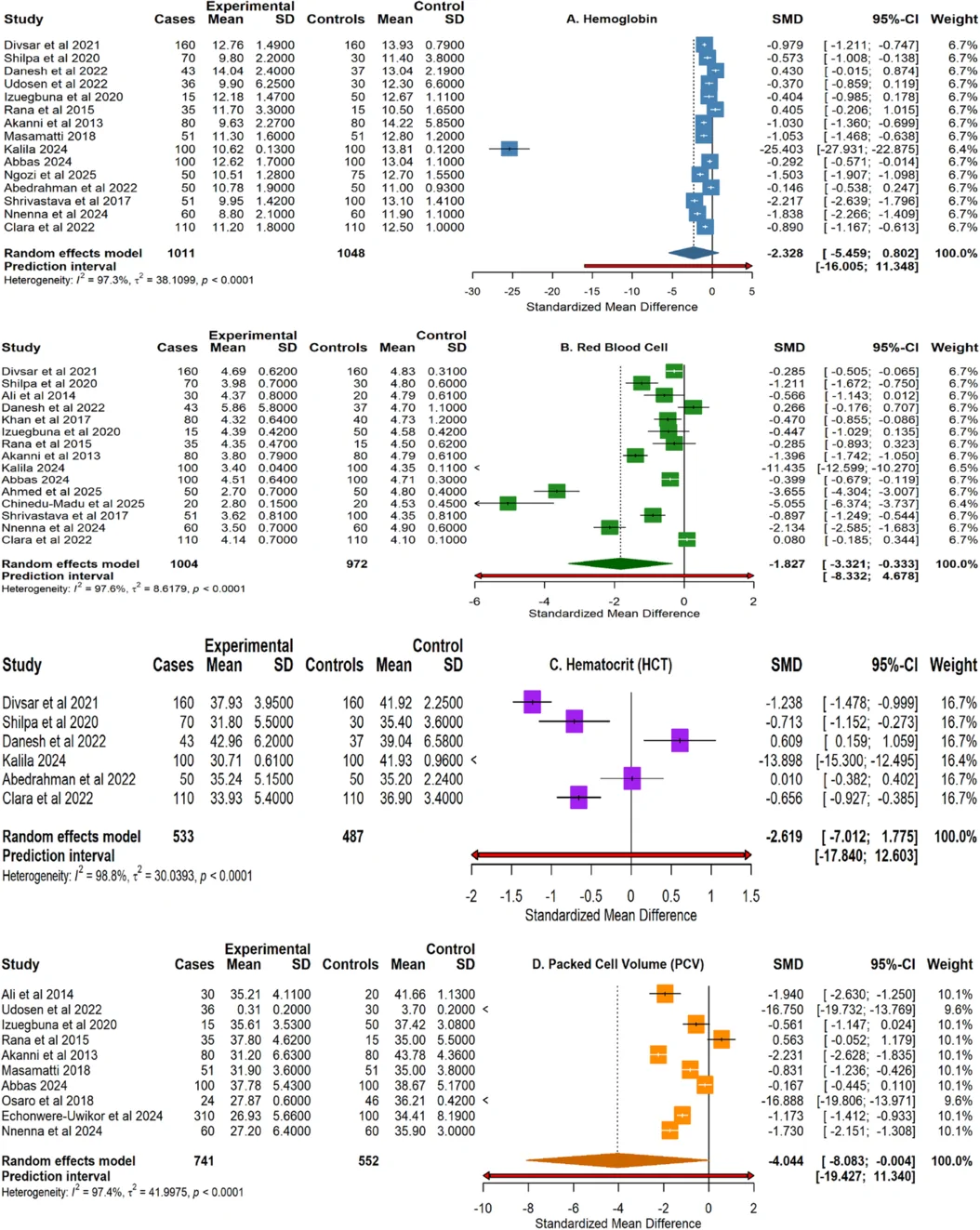
Forest plots showing pooled standardized mean differences (SMDs) for hemoglobin (Hb), red blood cell (RBC) count, hematocrit (HCT), and packed cell volume (PCV) comparing breast cancer patients (experimental group) with controls. Squares represent individual study effect sizes weighted by inverse variance; horizontal lines denote 95% confidence intervals. The diamond represents the pooled random‐effects estimate, and the red line indicates the prediction interval. Negative SMD values indicate lower hematological indices among breast cancer patients relative to controls. (A) Hemoglobin (HB), (B) Red Blood Cell (RBC), (C) Hematocrit (HCT), and (D) Packed Cell Volume (PCV).

### 3.6 Random‐Effects Meta‐Analysis of Red Cell Indices (MCV, MCH, and MCHC) in Breast Cancer Patients vs. Controls

MCV was significantly reduced in breast cancer patients (SMD = −1.150; 95% CI: −2.643 to −0.343; *I* ^2 ^= 97.7%). Similarly, MCH demonstrated a statistically significant decrease (SMD = −0.992; 95% CI: −2.187 to −0.204; *I* ^2 ^= 97.4%). In contrast, MCHC showed a non‐significant reduction (SMD = −1.091; 95% CI: −2.727 to 0.545; *I* ^2 ^= 97.7%). Heterogeneity was consistently very high across all red cell indices (Figure 3).

**Figure 3:**
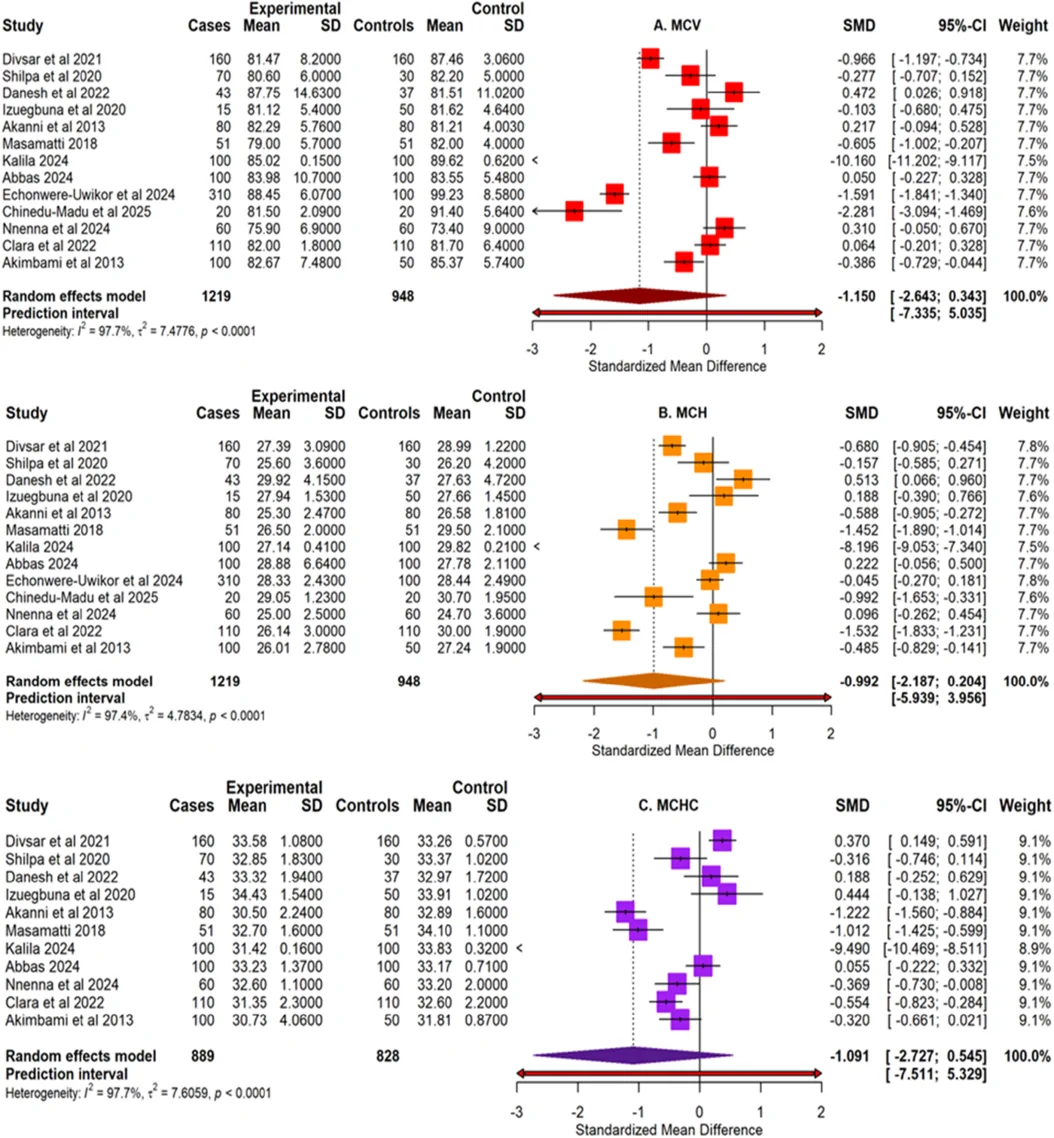
Forest plots showing pooled standardized mean differences (SMDs) for mean corpuscular volume (MCV) (A), mean corpuscular hemoglobin (MCH) (B), and mean corpuscular hemoglobin concentration (MCHC) (C) comparing breast cancer patients (experimental group) with controls. Squares represent individual study effect sizes weighted by inverse variance, with horizontal lines indicating 95% confidence intervals. Diamonds represent pooled random‐effects estimates, and red lines denote prediction intervals. Negative SMD values indicate lower red cell indices among breast cancer patients relative to controls.

### 3.7 Random‐Effects Meta‐Analysis of Total White Blood Cell Count and Differential Leukocyte Parameters in Breast Cancer Patients vs. Controls

Overall, total WBC count did not differ significantly between groups (SMD = 0.252; 95% CI: −1.773 to 2.276; *I* ^2 ^= 98.1%). In contrast, lymphocyte count was significantly reduced in breast cancer patients (SMD = −1.606; 95% CI: −3.191 to −0.020; *I* ^2 ^= 98.2%), whereas neutrophil count was significantly elevated (SMD = 1.109; 95% CI: 0.286–1.933; *I* ^2 ^= 97.0%). Monocyte count showed no statistically significant overall difference (SMD = 0.788; 95% CI: −0.285 to 1.860; *I* ^2 ^= 97.4%), and eosinophil count likewise demonstrated a non‐significant reduction (SMD = −2.326; 95% CI: −5.902 to 1.249; *I* ^2 ^= 96.8%). Notably, heterogeneity was consistently very high across all leukocyte parameters (Figure 4).

**Figure 4:**
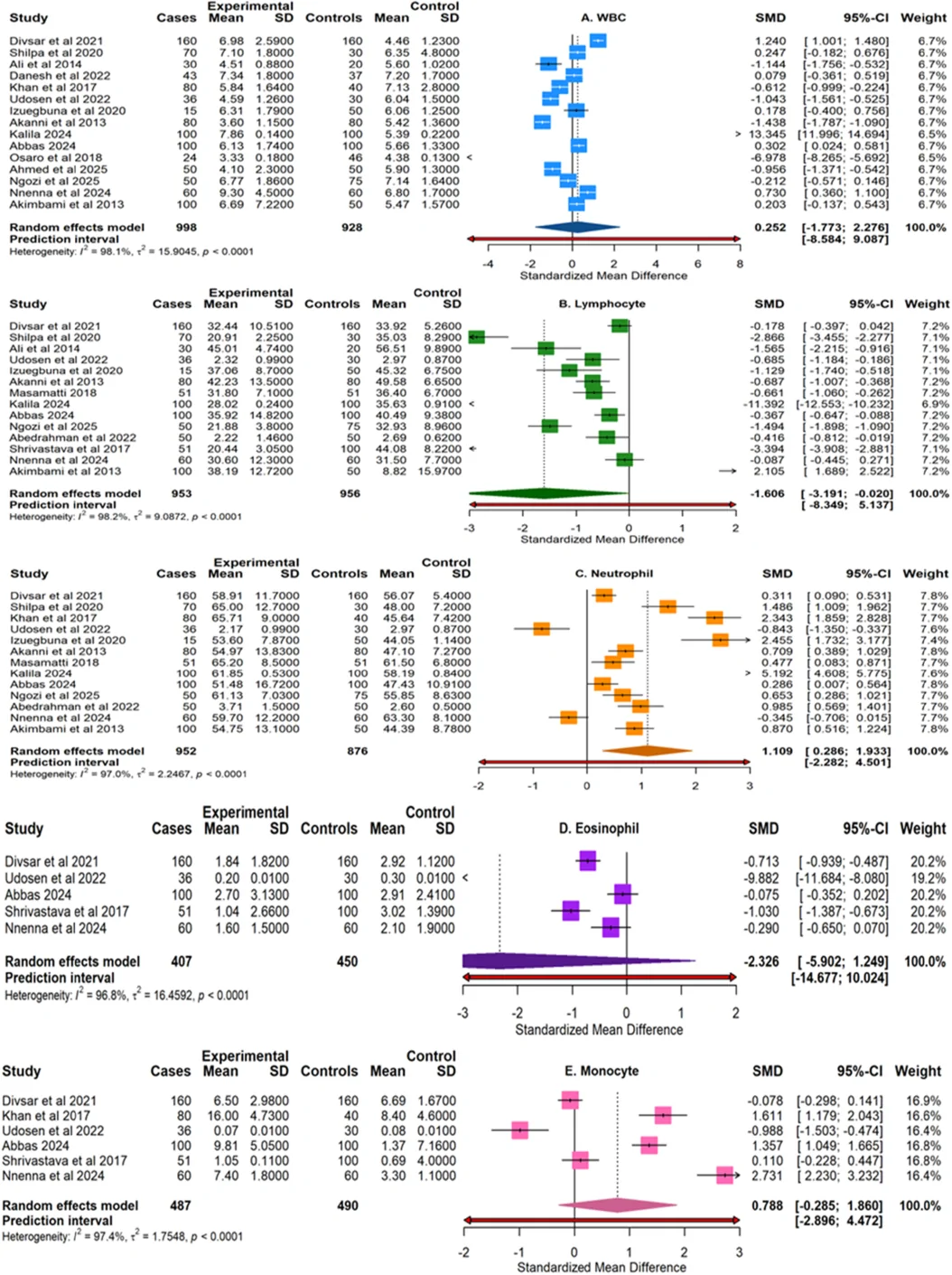
Forest plots showing pooled standardized mean differences (SMDs) for total white blood cell (WBC) count (A) and differential leukocyte parameters: lymphocyte (B), neutrophil (C), monocyte (E), and eosinophil (D) counts comparing breast cancer patients (experimental group) with controls. Squares represent individual study effect sizes weighted by inverse variance, with horizontal lines indicating 95% confidence intervals. Diamonds denote pooled random‐effects estimates, and red lines represent prediction intervals. Positive SMD values indicate higher leukocyte counts in breast cancer patients, while negative values indicate lower counts relative to controls.

### 3.8 Meta‐Analysis of Platelet Count and Mean Platelet Count Among Included Studies

Overall, platelet count was lower in breast cancer patients; however, the pooled effect did not reach statistical significance (SMD = −0.388; 95% CI: −1.102 to 0.326; *I* ^2 ^= 96.9%). In contrast, mean platelet volume was significantly elevated among breast cancer patients (SMD = 0.441; 95% CI: 0.073–0.808; *I* ^2 ^= 80.7%). While heterogeneity was considerable for both parameters, it was markedly higher for platelet count than for MPV (Figure 5).

**Figure 5:**
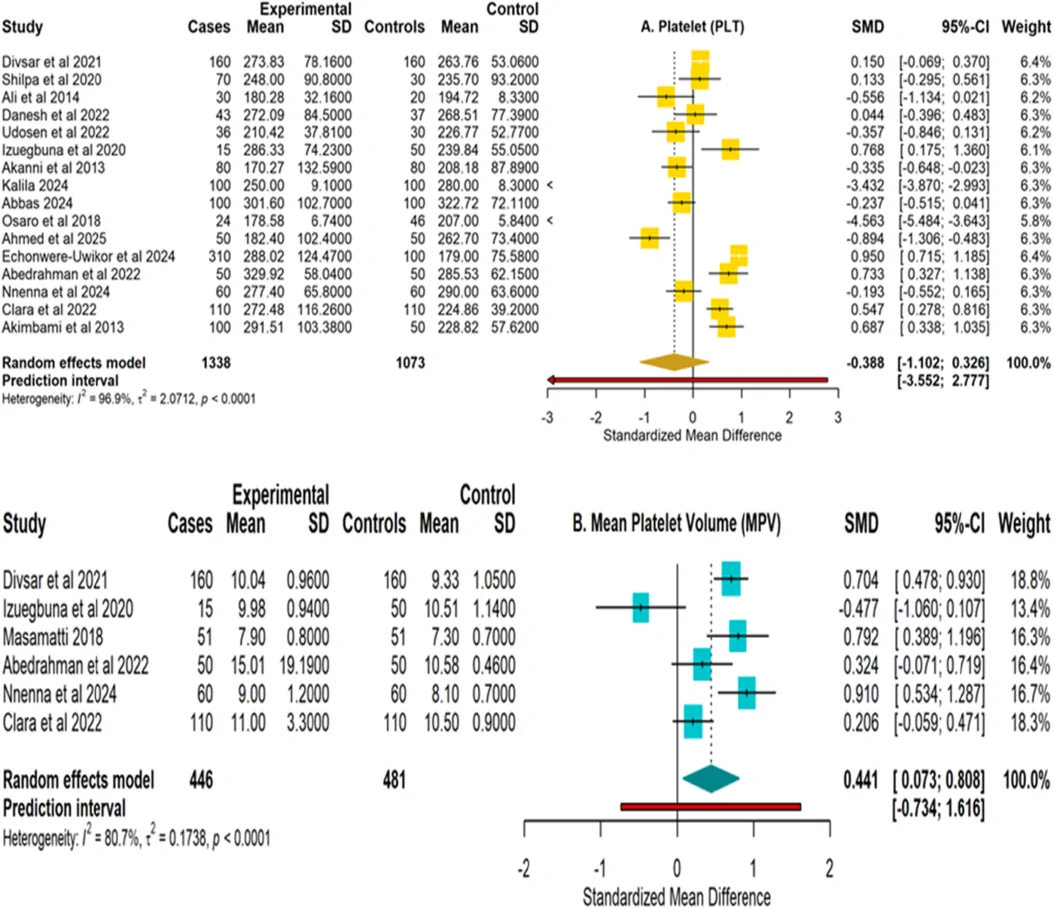
Forest plots showing pooled standardized mean differences (SMDs) for platelet count (PLT) (A) and mean platelet volume (MPV) (B) comparing breast cancer patients (experimental group) with controls. Squares represent individual study effect sizes weighted by inverse variance, horizontal lines denote 95% confidence intervals, diamonds indicate pooled random‐effects estimates, and red lines represent prediction intervals; negative SMD values indicate lower platelet indices, whereas positive values indicate higher indices in breast cancer patients relative to controls.

Table 6 presents the subgroup meta‐analysis of hematological parameters according to treatment status (before treatment vs. during chemotherapy). Among untreated patients, neutrophil count was significantly elevated (SMD = 1.559; 95% CI: 0.678–2.439; *p* = 0.001), and platelet count was likewise significantly higher (SMD = 0.436; 95% CI: 0.171–0.702; *p* = 0.001), whereas hemoglobin, RBC count, hematocrit, MCV, MCH, MCHC, WBC, lymphocyte count, and MPV showed no statistically significant differences. In contrast, during chemotherapy, MCV demonstrated a significant reduction (SMD = −1.11; 95% CI: −2.076 to −0.144; *p* = 0.024), while RBC count (SMD = −1.605; *p* = 0.119), packed cell volume (SMD = −6.908; *p* = 0.083), WBC (SMD = −2.95; *p* = 0.135), and lymphocyte count (SMD = −1.576; *p* = 0.082) exhibited larger negative effect sizes that did not reach statistical significance. Neutrophil, eosinophil, monocyte, and platelet counts during chemotherapy also showed non‐significant pooled effects. Across most subgroup analyses, statistical heterogeneity was negligible (*I* ^2 ^= 0%), likely reflecting the small number of studies within strata (Table 6).

**Table 6:** Subgroup meta‐analysis of hematological parameters by treatment status.

	Before treatment						Chemotherapy					
Parameter	Number of studies	References	SMD [95% CI]	*p* value	*I* ^2^	*p* value	Number of studies	References	SMD [95% CI]	*p* value	*I* ^2^	*p* value
Hemoglobin	4	[16, 34, 37, 39]	−0.372 [−0.94, 0.195]	0.199	0.009	0.000	4	[20, 22, 23, 32]	−0.823 [−1.908, 0.263]	0.138	0.009	0.000
RBC	4	[16, 34, 37, 39]	−0.315 [−0.967, 0.337]	0.343	0.009	0.000	3	[22, 27, 32]	−1.605 [−3.624, 0.414]	0.119	0.01	0.000
HCT	3	[16, 34, 39]	−0.262 [−1.097, 0.573]	0.538	0.009	0.000	0	—	—	—	—	—
PCV	0	—	—	—	—	—	5	[20, 22, 23, 26, 28]	−6.908 [−14.728, 0.913]	0.083	0.01	0.000
MCV	5	[16, 34, 35, 37, 39]	−0.051 [−0.345, 0.243]	0.735	0.006	0.029	2	[23, 28]	−1.11 [−2.076, −0.144]	0.024	0.009	0.000
MCH	5	[16, 34, 35, 37, 39]	−0.312 [‐1.013, 0.389]	0.383	0.009	0.000	2	[23, 28]	−0.735 [−2.114, 0.643]	0.296	0.01	0.000
MCHC	5	[16, 34, 35, 37, 39]	−0.159 [−0.501, 0.183]	0.362	0.007	0.006	0	—	—	—	—	—
WBC	4	[16, 35, 37, 39]	0.182 [−0.031, 0.394]	0.093	0	0.958	3	[20, 26, 27]	−2.95 [−6.815, 0.914]	0.135	0.01	0.000
Lymphocyte	3	[16, 34, 35, 37, 39]	−0.623 [−3.484, 2.238]	0.669	0.01	0.000	3	[20, 23, 32]	−1.576 [−3.351, 0.198]	0.082	0.01	0.000
Neutrophil	3	[16, 35, 37]	1.559 [0.678, 2.439]	0.001	0.009	0.000	2	[20, 23]	−0.173 [−1.467, 1.121]	0.793	0.009	0.000
Eosinophil	0	—	—	—	—	—	2	[20, 32]	−5.41 [−14.085, 3.264]	0.222	0.01	0.000
Monocyte	0	—	—	—	—	—	2	[20, 32]	−0.421 [−1.497, 0.654]	0.443	0.009	0.000
Platelet	5	[16, 35, 37, 39]	0.436 [0.171, 0.702]	0.001	0.005	0.068	4	[20, 26, 27, 28]	−1.187 [−3.473, 1.098]	0.309	0.01	0.000
MPV	2	[34, 37]	−0.084 [−0.745, 0.577]	0.804	0.008	0.037	0	—	—	—	—	—

*Note:* Effect sizes are presented as standardized mean differences (SMDs) with corresponding 95% confidence intervals (CIs). *I* ^2^ represents the percentage of variability attributable to between‐study heterogeneity.

### 3.9 Serum Cytokines in Breast Cancer Patients

From Table 7, the study by Hussain et al. [36] showed significantly higher levels of all three cytokines in the case group compared to the control group. Specifically, IL‐6 levels were 20.59 ± 5.04 pg/mL in cases versus 4.41 ± 1.01 pg/mL in controls (*p* = 0.001). IL‐10 levels were 8.79 ± 2.34 pg/mL in cases versus 2.83 ± 0.73 pg/mL in controls (*p* = 0.001). TNF‐α levels were 25.96 ± 6.92 pg/mL in cases versus 4.69 ± 1.58 pg/mL in controls (*p* = 0.001).

**Table 7:** Serum cytokines in breast cancer patients.

Hussain et al. [36] (pg/mL)	Control Mean ± SD	Cases Mean ± SD	*p* value
Total	20	60	
Interleukin 6	4.41 ± 1.01	20.59 ± 5.04	<0.001
Interleukin 10	2.83 ± 0.73	8.79 ± 2.34	<0.001
Tumor necrosis factor‐alpha	4.69 ± 1.58	25.96 ± 6.92	<0.001

## 4 Discussion

This meta‐analysis demonstrates that breast cancer in LMIC settings is associated with consistent and significant alterations in several hematological parameters, reflecting systemic changes that accompany malignancy and its treatment [6]. Among erythrocyte indices, red blood cell (RBC) count and packed cell volume (PCV) were significantly reduced, while hemoglobin and hematocrit showed non‐significant decreases with substantial heterogeneity. These findings align with previous reports describing anemia and reduced erythrocyte mass in breast cancer patients and likely reflect a combination of chronic inflammation, nutritional deficiencies, bone marrow suppression, and treatment‐related effects [40, 41]. Similar reductions in Hb, RBC, MCV, MCH, and HCT have been reported by Akanni [5] and more recently by Makkawi et al. [42], supporting the consistency of these observations across diverse populations. Importantly, while the direction of effect was uniform, the high heterogeneity observed suggests variability related to disease stage, treatment status, and population characteristics rather than uniform hematological impairment.

The significant reductions observed in mean corpuscular volume (MCV) and mean corpuscular hemoglobin (MCH) contrast with earlier findings and suggest a likely tendency toward microcytic and hypochromic patterns in some breast cancer populations. While previous studies have reported varying results for red cell indices [15, 39], the present findings may reflect differences in iron status, chronic inflammation, or regional nutritional patterns commonly encountered in LMICs [43, 44]. Reduced RBC indices have been associated with poorer clinical outcomes in breast cancer [40], reinforcing the clinical relevance of these routinely measured parameters as indicators of systemic burden rather than disease causation.

Alterations in leukocyte subsets were also prominent. Lymphocyte and eosinophil counts were significantly reduced, while monocyte counts were increased. These findings are consistent with earlier studies suggesting immune dysregulation and shifts in inflammatory cell populations in breast cancer patients [45]. Reduced lymphocyte counts may reflect immune exhaustion or treatment effects, whereas elevated monocytes have been associated with tumor‐associated inflammation and poorer prognostic profiles in breast cancer [46]. The marked reduction in eosinophils contrasts with findings by Poncin et al. [47], highlighting potential differences related to tumor subtype, immune context, or population‐specific factors. Collectively, these leukocyte alterations underscore the value of differential counts in capturing systemic immune changes associated with breast cancer.

Platelet indices also showed notable changes, with platelet count and mean platelet volume (MPV) significantly elevated prior to treatment. These findings are consistent with reports linking thrombocytosis and increased platelet activation to systemic inflammation and tumor burden [16]. Elevated MPV has been proposed as a marker of platelet reactivity and inflammatory status, which may reflect host–tumor interactions rather than direct tumor effects. As previously suggested, platelets may contribute to immune modulation and tumor cell protection within the circulation [48], although such mechanisms cannot be inferred from the present data.

Overall, the present findings reinforce the potential utility of routinely available hematological parameters as adjunctive indicators of systemic alterations in breast cancer patients, particularly in resource‐limited settings. However, the substantial heterogeneity observed across most analyses (*I* ^2 ^> 90%) warrants caution in interpreting the pooled estimates. This high degree of between‐study variability suggests that the magnitude of the observed differences may vary considerably across populations and clinical settings, potentially reflecting differences in study design, patient characteristics, disease stage, treatment exposure, and laboratory methods. Consequently, although the pooled results indicate overall patterns of hematological alterations associated with breast cancer, they should not be interpreted as uniform effects applicable to all LMIC populations. Furthermore, sensitivity and publication‐bias analyses, although useful for assessing the robustness of findings, cannot fully explain or eliminate this heterogeneity. The generalizability of the estimates may therefore be limited, particularly where the clinical and methodological characteristics of individual studies differ substantially. Future studies using standardized laboratory procedures and more consistent reporting of disease stage, treatment status, and other relevant clinical characteristics are needed to identify the sources of heterogeneity and improve the comparability of findings across settings.

The cytokine levels reported by Hussain et al. [36] demonstrated significant variations when compared to existing literature, providing valuable insights into the inflammatory landscape of breast cancer, particularly in LMICs. This study offers a unique perspective that highlights regional and possibly population‐specific differences in cytokine expression profiles.

For IL‐6, the observed levels in cases (20.59 ± 5.04 pg/mL) were markedly higher than those reported in a similar study conducted in a high‐income country. For instance, Ma et al. [49] recorded mean IL‐6 levels of approximately 12.3 pg/mL in breast cancer patients in China. This discrepancy could reflect variations in genetic, environmental, or lifestyle factors unique to LMIC populations. Additionally, differences in the stage of cancer at diagnosis, access to healthcare, and the prevalence of comorbidities such as infections and metabolic disorders common in LMICs may contribute to the elevated IL‐6 levels. The pro‐inflammatory cytokine IL‐6 is known to play a central role in tumor progression, angiogenesis, and immune modulation, and its heightened levels in this study underscore its potential utility as a biomarker for breast cancer in resource‐limited settings. Similarly, the TNF‐α levels reported in Hussain et al. study (25.96 ± 6.92 pg/mL) were significantly elevated compared to other studies. For example, Chen et al. [50] observed mean TNF‐α levels of 18.4 pg/mL in breast cancer patients in China, while Kozlowski et al. [51] reported levels of 16.2 pg/mL in a Polish cohort. TNF‐α is a key mediator of inflammation and has been implicated in cancer cell proliferation, invasion, and metastasis. The higher levels observed in Hussain et al. [36] study may reflect a greater systemic inflammatory response in LMIC populations, potentially driven by delayed cancer diagnoses and a higher burden of chronic inflammation from infectious diseases or other environmental exposures.

In contrast, IL‐10 levels reported by Hussain et al. (8.79 ± 2.34 pg/mL) were more consistent with existing literature. For instance, Chang et al. [52] reported comparable IL‐10 levels of approximately 7.9 pg/mL in breast cancer patients in China. However, even these levels were slightly elevated compared to other studies, suggesting nuanced differences in immune regulation. IL‐10 is an anti‐inflammatory cytokine that can have dual roles in cancer, promoting immune suppression while also potentially limiting inflammation‐induced tumor progression. Its levels in this study may reflect a compensatory anti‐inflammatory response to the heightened pro‐inflammatory cytokine milieu. Future studies should aim to validate these findings in larger cohorts and investigate their implications for personalized treatment approaches, particularly in resource‐limited settings where cytokine‐targeted therapies may hold promise as an affordable and effective option.

## 5 Conclusion

This meta‐analysis demonstrates that breast cancer patients in LMICs exhibit consistent alterations in hematological parameters and inflammatory cytokine profiles when compared with controls. Significant reductions in erythrocyte indices, alongside changes in leukocyte subsets and platelet parameters, reflect systemic immune and inflammatory perturbations associated with breast cancer and its management. Additionally, elevated pro‐inflammatory cytokines such as IL‐6 and TNF‐α, together with altered IL‐10 levels, highlight a distinct inflammatory milieu that may vary across populations. Collectively, these findings support the potential role of routine complete blood count indices and selected cytokines as adjunctive markers for monitoring disease burden and immune status in resource‐limited settings.

### 5.1 Strengths and Limitations

The key strength of this study lies in its focus on LMIC populations, addressing an important gap by synthesizing evidence from resource‐limited settings where the burden of breast cancer is increasing and access to advanced diagnostics may be limited. However, several limitations must be acknowledged. Substantial heterogeneity was observed across included studies, likely reflecting differences in study design, sample size, patient characteristics, disease stage, and treatment status. Importantly, several studies did not clearly report how breast cancer diagnosis was confirmed (e.g., histopathological verification), which may affect comparability and methodological rigor. In addition, incomplete reporting of clinical variables such as chemotherapy duration, tumor subtype, and staging limited stratified analyses. The relatively small number of cytokine studies further restricted quantitative synthesis of inflammatory markers.

### 5.2 Recommendations

Future studies should prioritize well‐designed, multicenter prospective investigations in LMICs with standardized reporting of hematological and cytokine parameters, stratified by disease stage and treatment status. Integrating routine hematological indices with cytokine profiling may enhance risk stratification and disease monitoring, particularly where advanced diagnostics are unavailable. Further research is also warranted to explore longitudinal changes in these biomarkers and their potential integration into cost‐effective clinical monitoring frameworks tailored to resource‐limited healthcare systems.

## Author Contributions

**Richard Vikpebah Duneeh:** conceptualization, writing – review and editing, validation, methodology. **Otchere Addai‐Mensah:** writing – review and editing, supervision, methodology. **Benedict Sackey:** writing – review and editing, methodology. **Ernest Adankwah:** writing – review and editing, conceptualization, supervision. **Edward Yaw Afriyie:** methodology, formal analysis, visualization. **Veronica Agyemang:** data curation, methodology. **Precious Kwablah Kwadzokpui:** methodology, formal analysis, visualization. **Emmanuel Ativi:** writing – original draft, formal analysis. **Mercy Adzo Klugah:** validation, writing – review and editing, supervision, data curation. **Francis Agyei Amponsah:** writing – review and editing, writing – original draft, visualization, validation. **Kenneth Ablordey:** formal analysis, writing – original draft, data curation.

## Funding

The authors have nothing to report.

## Ethics Statement

The authors have nothing to report.

## Conflicts of Interest

The authors declare no conflicts of interest.

## Transparency Statement

The lead author, Kenneth Ablordey, affirms that this article is an honest, accurate, and transparent account of the study being reported; that no important aspects of the study have been omitted; and that any discrepancies from the study as planned (and, if relevant, registered) have been explained.

## Supporting information


Supporting File

## Data Availability

The datasets used in this study are publicly available in international databases (PubMed, Web of Science, Scopus, Google Scholar) and can be accessed using the search terms provided in the Methods section.

## References

[1] F. Bray , J. Ferlay , I. Soerjomataram , R. L. Siegel , L. A. Torre , and A. Jemal , “Global Cancer Statistics 2018: GLOBOCAN Estimates of Incidence and Mortality Worldwide for 36 Cancers in 185 Countries,” CA: A Cancer Journal for Clinicians 68 (2018): 394–424, 10.3322/caac.21492. 30207593

[2] D. Adeloye , O. Y. Sowunmi , W. Jacobs , et al., “Estimating the Incidence of Breast Cancer in Africa: A Systematic Review and Meta‐Analysis,” Journal of Global Health 8 (2018): 010419, 10.7189/jogh.08.010419. PMC5903682 29740502

[3] I.‐H. Seo and Y.‐J. Lee , “Usefulness of Complete Blood Count (CBC) to Assess Cardiovascular and Metabolic Diseases in Clinical Settings: A Comprehensive Literature Review,” Biomedicines 10 (2022): 2697, 10.3390/biomedicines10112697. PMC9687310 36359216

[4] H. Sun , C. Q. Yin , Q. Liu , F. Wang , and C. H. Yuan , “Clinical Signiﬁcance of Routine Blood Test‐Associated Inflammatory Index in Breast Cancer Patients,” Medical Science Monitor: International Medical Journal of Experimental and Clinical Research 23 (2017): 5090–5095, 10.12659/MSM.906709. PMC5667583 29069071

[5] O. Akanni , A. S. Oguntola , M. L. Adeoti , and O. Agodirin , “Haematological Parameters in Female Breast Cancer Patients in South Western Nigeria,” International Journal of Medical and Health Sciences 2 (2013): 2277–4505.

[6] M. Aynalem , N. Adem , F. Wendesson , et al., “Hematological Abnormalities Before and After Initiation of Cancer Treatment Among Breast Cancer Patients Attending at the University of Gondar Comprehensive Specialized Hospital Cancer Treatment Center,” PLoS One 17 (2022): e0271895, 10.1371/journal.pone.0271895. PMC9359539 35939445

[7] Chinedu‐Madu , C. A. Onyenze , and O. Aloy‐Amadi , “Assessment of Red Blood Cell Count and Red Cell Indices in Patients Diagnosed With Breast Cancer at Federal Teaching Hospital, Owerri Nigeria,” International Journal of Clinical and Medical Case Reports 4, no. 3 (2025): 1–5, 10.31579/2834-8664/070.

[8] E. Bryer and D. Henry , “Chemotherapy‐Induced Anemia: Etiology, Pathophysiology, and Implications for Contemporary Practice,” International Journal of Clinical Transfusion Medicine 6 (2018): 21–31, 10.2147/IJCTM.S187569.

[9] A. Maccio , C. Madeddu , G. Gramignano , et al., “The Role of Inflammation, Iron, and Nutritional Status in Cancer‐Related Anemia: Results of a Large, Prospective, Observational Study,” Haematologica 100 (2015): 124–132, 10.3324/haematol.2014.112813. PMC4281325 25239265

[10] F. R. Greten and S. I. Grivennikov , “Inflammation and Cancer: Triggers, Mechanisms and Consequences,” Immunity 51 (2019): 27–41, 10.1016/j.immuni.2019.06.025. PMC6831096 31315034

[11] V. Camacho , V. Kuznetsova , and R. S. Welner , “Inflammatory Cytokines Shape an Altered Immune Response During Myeloid Malignancies,” Frontiers in Immunology 12 (2021): 772408, 10.3389/fimmu.2021.772408. PMC8595317 34804065

[12] S. Kany , J. T. Vollrath , and B. Relja , “Cytokines in Inflammatory Disease,” International Journal of Molecular Sciences 20 (2019): 6008, 10.3390/ijms20236008. PMC6929211 31795299

[13] C. H. Yip , I. Buccimazza , M. Hartman , S. V. S. Deo , and P. S. Y. Cheung , “Improving Outcomes in Breast Cancer for Low and Middle Income Countries,” World Journal of Surgery 39 (2015): 686–692, 10.1007/s00268-014-2859-6. 25398564

[14] G. Wells , B. Shea , D. O'connell , J. Peterson , V. Welch , and M. Losos , “The Newcastle–Ottawa Scale (NOS) for Assessing the Quality of Nonrandomised Studies in Meta‐Analyses [Internet],” published 2018, https://wwwohri.ca/programs/clinical_epidemiology/oxford.asp.

[15] B. Divsalar , P. Heydari , G. Habibollah , and G. Tamaddon , “Hematological Parameters Changes in Patients With Breast Cancer,” Clinical Laboratory 67 (2021): 1832–1840, 10.7754/Clin.Lab.2020.201103. 34383417

[16] M. D. Shilpa , R. Kalyani , and P. N. Sreeramulu , “Prognostic Value of Pre‐Treatment Routine Hematological Parameters in Breast Carcinoma: Advantageous or Deleterious?,” Biomedical Research and Therapy 7 (2020): 3916–3920, 10.15419/bmrat.v7i8.621.

[17] L. O. Ali , “Study Effect of Breast Cancer on Some Hematological and Biochemical Parameters in Babylon Province, Iraq,” IOSR Journal of Pharmacy and Biological Sciences 9 (2014): 20–24, 10.9790/3008-09352024.

[18] H. Danesh , N. Ziamajidi , S. A. Mesbah‐Namin , N. Nafisi , and R. Abbasalipourkabir , “Association Between Oxidative Stress Parameters and Hematological Indices in Breast Cancer Patients,” International Journal of Breast Cancer 2022 (2022): 1–8, 10.1155/2022/1459410. PMC9550463 36225290

[19] S. Khan , S. Khoso , S. Memon , A. Adeel , and G. Nabi , “Study of Some Haematological Parameters as Biomarker for Breast Cancer Populations of Sindh,” Sindh University Research Journal 49 (2017): 23–28, https://sujo.usindh.edu.pk/index.php/SURJ/article/view/2951/2188.

[20] J. E. Udosen , E. C. Akwiwu , D. U. Akpotuzor , and J. O. Akpotuzor , “Some Haematological Parameters of Breast Cancer Patients Accessing Therapy at University of Calabar Teaching Hospital, Calabar Nigeria,” Journal of Medical Laboratory Science 7 (2022): 89–93, https://www.ajol.info/index.php/sokjmls/article/view/226508.

[21] O. H. O. Ogochukwu , S. A. Olatoke , O. S. Agodirin , and O. O. Izuegbuna . Open Access Journal 31 (2020): 110–131, https://www.researchgate.net/publication/348389718_Haemogram_Pattern_and_Khorana_Score_of_Breast_Cancer_Patients_in_A_Tertiary_Centre_in_Nigeria_TMJ.

[22] A. P. S. Rana , M. Kaur , B. Zonunsanga , A. Puri , and A. S. Kuka , “Preoperative Peripheral Blood Count in Breast Carcinoma: Predictor of Prognosis or a Routine Test,” International Journal of Breast Cancer 2015 (2015): 1–5, 10.1155/2015/964392. PMC4677208 26697229

[23] S. S. Masamatti , “Hematological Parameters in Pre Chemotherapy Breast Cancer Patients in a Tertiary Care Centre,” IP Journal of Diagnostic Pathology and Oncology 3 (2020): 237–240, 10.18231/2581-3706.2018.0049.

[24] A. A. S. Kalila , “Evaluate the Alterations in Hematological Parameters Among Breast Cancer Patients at the Sabratha National Cancer Institute in Western Libya,” IP Journal of Diagnostic Pathology and Oncology 8 (2014): 2490.

[25] A. B. Abbas , S. Al‐Gamei , A. Naser , et al., “Comparison of Hematological Parameters and the Associated Factors Among Women With and Without Breast Cancer: A Case‐Control Study,” Breast Cancer: Targets and Therapy 16 (2024): 877–885, 10.2147/BCTT.S497313. PMC11645957 39678025

[26] E. Osaro , M. E Chinoye , R. Michael , E. Tosan , and A. T. Charles , “Some Full Blood Count Parameters Among Women of African Descent With Breast Cancer in Sokoto, North Western Nigeria,” Journal of Pregnancy and Reproduction 2 (2018): 1–6, 10.15761/JPR.1000129.

[27] H. A. F. Ahmed , S. H. M. Ahmed , S. D. A. Mohamed , et al., “Evaluation of Hematological Parameters Pre and Post Chemotherapy in Breast Cancer Patients at the National Cancer Institute in Sudan at Khartoum State, Sudan,” Journal of Cancer and Tumor International 15 (2025): 74–80, 10.9734/jcti/2025/v15i1284.

[28] B. E. Echonwere‐Uwikor and F. K. Uwikor , “Association Between Haematological Parameters and Different Stages of Breast Cancer for Patients Undergoing Chemotherapy in Rivers State University Teaching Hospital, Port Harcourt, Nigeria,” Otuoke Journal of Medical Laboratory Science 1 (2024): 65–69.

[29] A. R. Ngozi , N. Harrison , E. Ukamaka , N. I. Godwin , C. Obasi , and M. Gertrude , “Evaluation of Some Hematological Parameters in Breast Cancer Patients Attending Oncology Clinic at American Cancer Hospital, Iho, Imo State,” Journal of Biomedical Research and Clinical Advancements 2 (2025): 34.

[30] S. K. Abedalrahman , I. M. Zahwan , A. A. Ali , and M. M. Jassem , “Predicting Breast Cancer Risk Using Simple Blood Parameters,” Neuro Quantology 20 (2022): 3517–3523.

[31] R. P. Storph , F. N. Ghartey , and R. K. D. Ephraim , et al., “The Effect of Chemotherapy on Clinical, Haematological and Biochemical Profile in Breast Cancer Patients Undergoing Chemotherapy at Cape Coast Teaching Hospital: A Longitudinal Study,” Journal of Cancer and Tumor International 9, no. 2 (2019): 1–9, 10.9734/jcti/2019/v9i230104.

[32] S. Shrivastava , N. Singh , A. K. Nigam , S. S. Chandel , R. Shrivastava , and S. Kumar , “Comparative Study of Hematological Parameters Along With Effect of Chemotherapy and Radiotherapy in Different Stages of Breast Cancer,” International Journal of Research in Medical Sciences 5 (2016): 311, 10.18203/2320-6012.ijrms20164569.

[33] K. U. Nnenna , K.‐E. Su , N. A. Okoye , and E. M. Eze , “Full Blood Count and Cancer Antigen 15.3 Levels of Breast Cancer Subjects in Umuahia, Abia State, Nigeria,” International Journal of Research and Reports in Hematology 7 (2024): 23–32.

[34] N. S. Clara , N. C. Ibeh , F. O. Ugwuene , et al., “Use the Complete Blood Counts and ESR as Biomarkers Prognostic Tool in Breast Cancer Patients Attending Surgery Department at Esut Teaching Hospital, Parklane Enugu Nigeria,” Journal of Pharmaceutical Research International 34 (2022): 6–29, 10.9734/jpri/2022/v34i39A36230.

[35] A. Akinbami , A. Popoola , A. Adediran , et al., “Full Blood Count Pattern of Pre‐Chemotherapy Breast Cancer Patients in Lagos,” Nigeria 49 (2013): 23–28. PMC3762228 24009939

[36] A. M. Hussain , A. H. Ali , and H. L. Mohammed , “Estimation Serum Levels of Some Cytokines in the Breast Cancer of Iraqi Women,” International Journal Pharmacology Research 12 (2020): 1034–1038, 10.31838/IJPR/2020.12.02.0156.

[37] O. O. Izuegbuna , H. O. Olawumi , S. A. Olatoke , and O. S. Agodirin , “Haemogram Pattern and Khorana Score of Breast Cancer Patients in A Tertiary Centre in Nigeria,” Tanzania Medical Journal 31 (2020): 110–131.

[38] M. M. Rana , M. S. Siddiqee , M. N. Sakib , and M. R. Ahamed , “Assessing AI Adoption in Developing Country Academia: A Trust and Privacy‐Augmented UTAUT Framework,” Heliyon [Internet] 10 (2024): 964392, 10.1016/j.heliyon.2024.e37569. PMC11417232 39315142

[39] H. Danesh , N. Ziamajidi , S. A. Mesbah‐Namin , N. Nafisi , and R. Abbasalipourkabir , “Association Between Oxidative Stress Parameters and Hematological Indices in Breast Cancer Patients,” International Journal of Breast Cancer 2022 (2022): 1–8, 10.1155/2022/1459410. PMC9550463 36225290

[40] F. M. S. Muthanna , M. Karuppannan , E. Abdulrahman , S. Uitrakul , B. A. H. Rasool , and A. H. Mohammed , “Prevalence and Associated Factors of Anemia Among Breast Cancer Patients Undergoing Chemotherapy: A Prospective Study,” Advances in Pharmacological and Pharmaceutical Sciences 2022 (2022): 7611733, 10.1155/2022/7611733. PMC9023199 35464620

[41] E. I. Obeagu , “Mentzer Index in Breast Cancer: Insights into Anemia and Tumor Progression—A Narrative Review,” Medicine 104 (2025): e45114, 10.1097/MD.0000000000045114. PMC12499847 41054087

[42] M. Makkawi , L. Alasiri , and S. Alasmari , “Assessing the Prevalence of Breast Tumors and Associated Abnormalities in Hematological and Coagulation Parameters in the Asir Region, Saudi Arabia,” Annals of Clinical and Laboratory Science 55 (2025): 373–379. 40750235

[43] E. Araujo Costa and J. de Paula Ayres‐Silva , “Global Profile of Anemia During Pregnancy Versus Country Income Overview: 19 Years Estimative (2000–2019),” Annals of Hematology 102 (2023): 2025–2031, 10.1007/s00277-023-05279-2. PMC10344983 37233775

[44] M. Bayati , E. Arkia , and M. Emadi , “Socio‐Economic Inequality in the Nutritional Deficiencies Among the World Countries: Evidence From Global Burden of Disease Study 2019,” Journal of Health, Population, and Nutrition 44 (2025): 8, 10.1186/s41043-025-00739-z. PMC11731139 39806471

[45] X. Jiang and D. J. Shapiro , “The Immune System and Inflammation in Breast Cancer,” Molecular and Cellular Endocrinology 382 (2014): 673–682, 10.1016/j.mce.2013.06.003. PMC4919022 23791814

[46] B. Azab , V. R. Bhatt , J. Phookan , et al., “Usefulness of the Neutrophil‐to‐Lymphocyte Ratio in Predicting Short‐ and Long‐Term Mortality in Breast Cancer Patients,” Annals of Surgical Oncology 19 (2012): 217–224, 10.1245/s10434-011-1814-0. 21638095

[47] A. Poncin , C. E. Onesti , C. Josse , et al., “Immunity and Breast Cancer: Focus on Eosinophils,” Biomedicines 9 (2021): 1087, 10.3390/biomedicines9091087. PMC8470317 34572273

[48] H. Takeuchi , M. Abe , Y. Takumi , et al., “The Prognostic Impact of the Platelet Distribution Width‐to‐Platelet Count Ratio in Patients With Breast Cancer,” PLoS One 12 (2017): e0189166, 10.1371/journal.pone.0189166. PMC5720724 29216259

[49] Y. Ma , Y. Ren , Z. J. Dai , C. J. Wu , Y. H. Ji , and J. Xu , “IL‐6, IL‐8 and TNF‐α Levels Correlate With Disease Stage in Breast Cancer Patients,” Advances in Clinical and Experimental Medicine: Official Organ Wroclaw Medical University 26 (2017): 421–426, 10.17219/ACEM/62120. 28791816

[50] Y. Chen , S. Zhang , Q. Wang , and X. Zhang , “Tumor‐Recruited M2 Macrophages Promote Gastric and Breast Cancer Metastasis via M2 Macrophage‐Secreted CHI3L1 Protein,” Journal of Hematology & Oncology 10 (2017): 1–13, https://link.springer.com/article/10.1186/s13045-017-0408-0. 10.1186/s13045-017-0408-0 PMC5286803 28143526

[51] L. Kozłowski , I. Zakrzewska , P. Tokajuk , and M. Z. Wojtukiewicz , “Concentration of Interleukin‐6 (IL‐6), Interleukin‐8 (IL‐8) and Interleukin‐10 (IL‐10) in Blood Serum of Breast Cancer Patients,” Roczniki Akademii Medycznej w Bialymstoku (1995) 48 (2003): 82–84. 14737948

[52] C. M. Chang , H. Y. P. Lam , H. J. Hsu , and S. J. Jiang , “Interleukin‐10: A Double‐Edged Sword in Breast Cancer,” Tzu Chi Medical Journal 33 (2021): 203–211, 10.4103/TCMJ.TCMJ_162_20. PMC8323643 34386356

