# Supplementary material for: Hematological Profiles and Serum Cytokine Levels in Patients With Breast Cancer in Low‐ and Middle‐Income Countries: A Systematic Review and Meta‐Analysis of Current Evidence
Source: Health Sci Rep. 2026 Oct 27;9(11):e73451. doi: 10.1002/hsr2.73451 (PMC13711481; doi:10.1002/hsr2.73451)
Supplement: Supplementary file 1 [file HSR2-9-e73451-s001.docx]

**Detailed Country Classification with World Bank Income Groups**

| Country | Studies (n) | GNI per Capita (USD)* | World Bank Income Group | Classification Criteria | Verification Source |
| --- | --- | --- | --- | --- | --- |
| Ethiopia | 1 | < $1,135 | Low-income | GNI ≤ $1,135 | World Bank 2024 |
| Yemen | 1 | < $1,135 | Low-income | GNI ≤ $1,135 | World Bank 2024 |
| Sudan | 1 | < $1,135 | Low-income | GNI ≤ $1,135 | World Bank 2024 |
| India | 5 | $1,136 - $4,465 | Lower-middle-income | $1,136 ≤ GNI ≤ $4,465 | World Bank 2024 |
| Nigeria | 8 | $1,136 - $4,465 | Lower-middle-income | $1,136 ≤ GNI ≤ $4,465 | World Bank 2024 |
| Pakistan | 1 | $1,136 - $4,465 | Lower-middle-income | $1,136 ≤ GNI ≤ $4,465 | World Bank 2024 |
| Ghana | 1 | $1,136 - $4,465 | Lower-middle-income | $1,136 ≤ GNI ≤ $4,465 | World Bank 2024 |
| Libya | 1 | $4,466 - $13,845 | Upper-middle-income | $4,466 ≤ GNI ≤ $13,845 | World Bank 2024 |
| Iraq | 4 | $4,466 - $13,845 | Upper-middle-income | $4,466 ≤ GNI ≤ $13,845 | World Bank 2024 |
| Iran | 2 | $4,466 - $13,845 | Upper-middle-income | $4,466 ≤ GNI ≤ $13,845 | World Bank 2024 |

*GNI per capita figures are based on the World Bank Atlas method for the 2024 fiscal year.

**Reference:**
13. World Bank. World Bank Country and Lending Groups – 2024 Fiscal Year. Washington, DC: The World Bank; 2024. Available from: <https://datahelpdesk.worldbank.org/knowledgebase/articles/906519-world-bank-country-and-lending-groups>
